# Case report: Retrieval of knotted ureteric stent causing obstructive urosepsis

**DOI:** 10.1016/j.eucr.2023.102316

**Published:** 2023-01-04

**Authors:** G.N. Weeratunga, L. Yuan, O. Yassaie, P. Caswell-Smith

**Affiliations:** Wellington Hospital, Capital & Coast, Te Whatu Ora, New Zealand

## Abstract

Knotting of ureteric stents is a rare complication, with only 30 cases being described and can be vexing to manage. A few strategies have been described in the literature in a very limited collection of case reports.

Currently there are no reports of successful percutaneous removal in the setting of obstructive urosepsis. Herein we discuss our approach to this case, where a ureteric stent was placed for elective ureteric identification for an Abdominal Aortic Aneurysm repair and removed successfully with an antegrade approach without the use of nephroscopy.

## 1 Introduction

Ureteric stent placement is a common procedure performed for a range of indications including internal drainage of the upper urinary tract for obstruction and to aid ureteric identification in complex abdominal surgery. Knotting of urethral stents is a rare complication, hindering their routine removal by flexible cystoscopy under local anesthesia. Fewer than 30 case reports have been published with a variety of techniques described to achieve stent retrieval.^1^

Herein we discuss a case of a knotted stent with subsequent obstructive urosepsis. The stent was removed via a percutaneous antegrade approach. To our knowledge, this is the first reported case of percutaneous stent removal in the context of obstructive urosepsis.

## 2 Case report

A 73-year-old male underwent routine bilateral ureteric stent placement to help identify the ureters prior to an elective open AAA repair. Accurate stent positioning was confirmed on image intensifier.

The patient was catheter dependent from BPH and had Granulomatosis with polyangiitis (GPA) for which he was on methotrexate and prednisone for immunosuppression. Initially a plan was made to remove the stents six weeks post operatively to minimize the risk of multiple instrumentations of the lower urinary tract, immediately after AAA repair to reduce the chance of a significant urinary tract infection. Unfortunately, the patient experienced bothersome hematuria once ambulant post operatively as the patient was also on rivaroxaban for stroke prophylaxis in the setting of atrial fibrillation. Given this outpatient-based stent removal was performed on post-operative day eight.

**Fig. 1:**
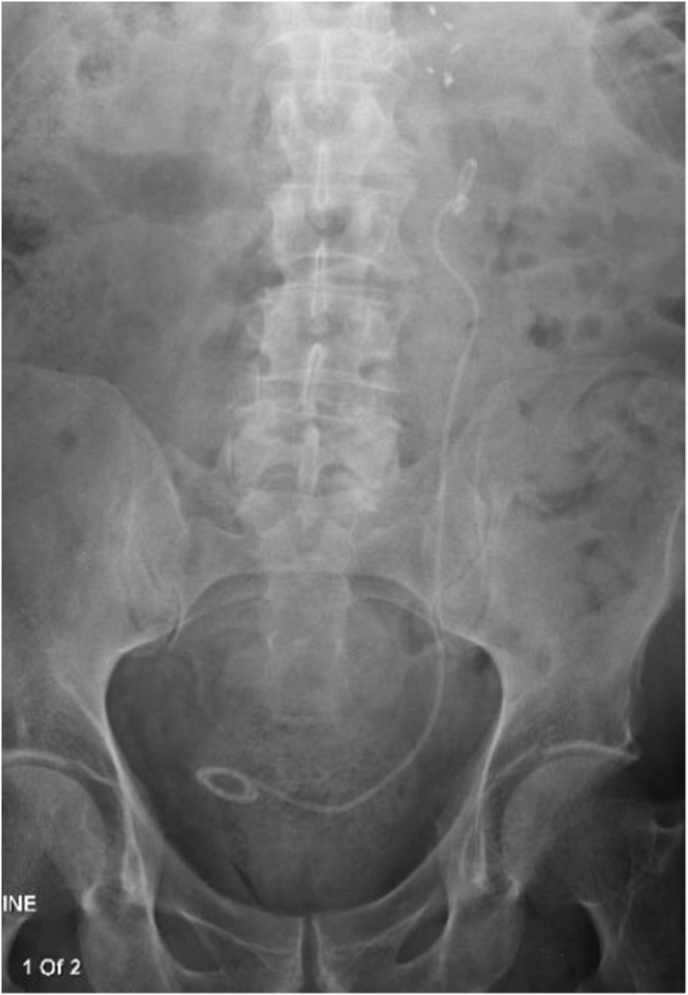
Left knotted ureteric stent on abdominal x-ray.

The right sided stent was removed without complication using a flexible cystoscope, under local lidocaine lubrication, in the outpatient clinic. An attempt at stent removal on the left was met with resistance and the procedure was abandoned. Xray demonstrated a knot at the proximal coil of the stent, which was pulled down into the proximal ureter (Fig. 1.). The patient was waitlisted for surgery to remove the stent under a general anesthetic. In the interim, the patient was admitted acutely with urosepsis. CT demonstrated hydronephrosis and perinephric stranding suggestive of obstructive urosepsis.

**Fig. 2:**
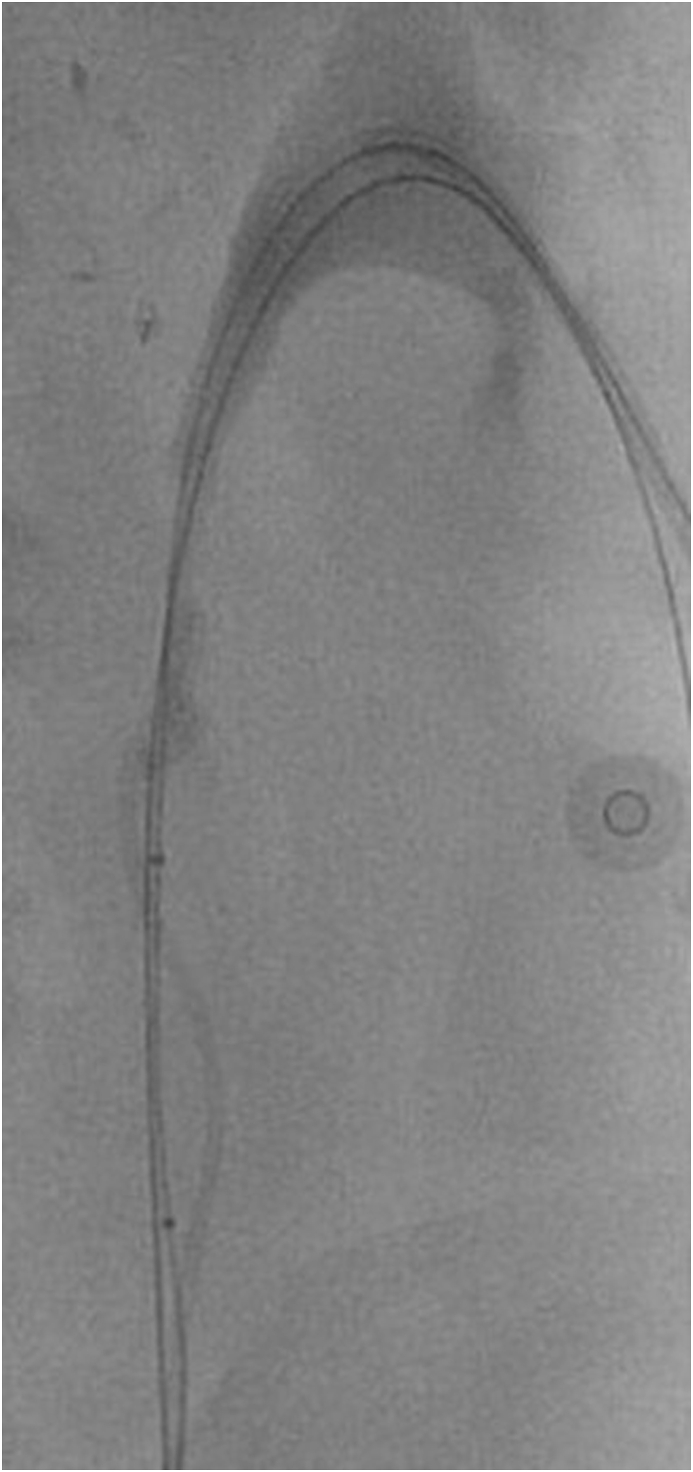
Attempted retrieval of knotted stent with 6 × 40mm PET balloon.

**Fig. 3:**
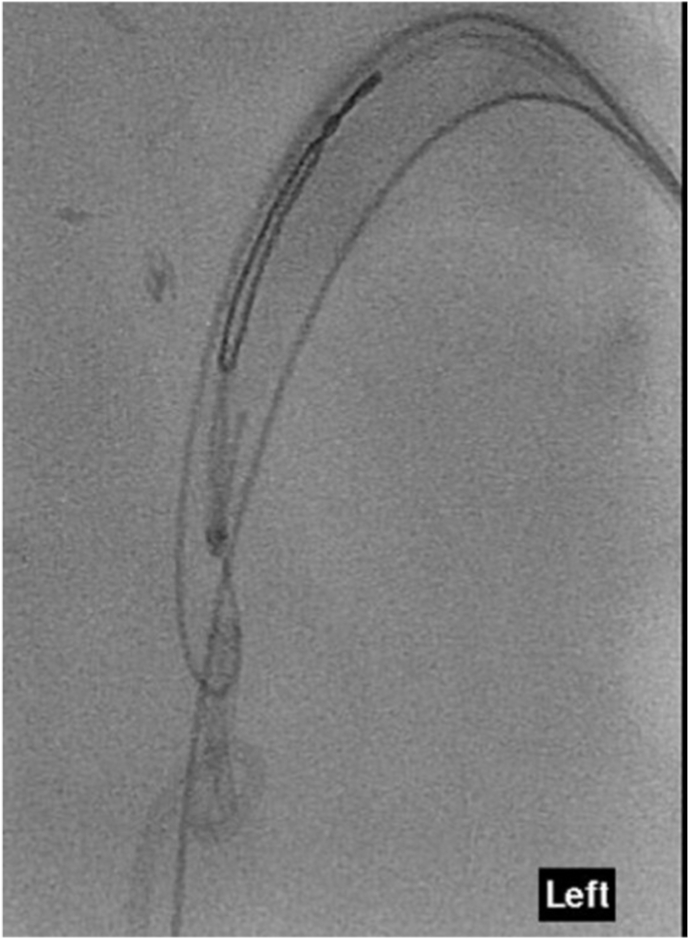
Retrieval of knotted stent with snare-wire technique.

Interventional Radiology was consulted for an antegrade approach. Using ultrasound guidance, a lower pole calyx was punctured, and access secured with an 8-French sheath. A soft 0.035 angled Glidewire(Terumo), though a 4Fr Bern catheter, was passed distal to the knotted stent and an Amplatz wire was exchanged into the ureter as a safety/support wire. Initial attempts to snare the stent from above and then the knot itself from underneath using a gooseneck snare were unsuccessful as the knot was firmly wedged against the ureteric wall. An attempt to dislodge the knotted stent up into the renal pelvis by inflating a 6 × 40mm balloon distal to the knot and pulling it up was also unsuccessful (Fig. 2.) Finally, a “loop-snare” technique was utilized by threading a Glidewire through the superior loop of the knot, snaring the wire and retracting the snared wire which ultimately untied the knot and retrieved the stent (Fig. 3.). An 8-French nephrostomy tube was left in situ for drainage. Urine and blood cultures revealed Candida Albicans. The patient recovered with a temporary nephrostomy tube and antifungal treatment.

## 3 Discussion

Ureteric stent insertion following ureteroscopic stone treatment remains a commonly performed procedure, with rates of stent usage as high as 70% following ureteroscopy. Although rare, removal of knotted stents poses a significant technical challenge with potential complications, including significant ureteric injury and loss of renal unit.^2^

Since its first description in 1989 by Groenewald, less than 30 reports are described in our MEDLINE literature search using “knotted ureteric stent”. Knotted stents almost always occur within the proximal ureter.^3^, ^4^, ^5^ 2 cases of mid ureteric knots and 1 case of distal ureteric knot are also described.

Although no causative factors have been established, it has been hypothesized that excessive stent length and coiling, either from multi-length ureteric stents or anatomical factors, may be a predisposing factor for stent knotting.^3^ Stent diameters from 4.7Fr-7Fr have been reported in the literature and it did not appear this was a significant risk factor.

Only one case of repeat stent knotting in a single patient is reported. Interestingly, the knot spontaneously untied itself after strong Valsalva during difficult intubation. The stent was then retrieved via extraction strings. Ahmadi et al. reported a case of bilateral knot formation in bilateral stents in a single patient.^2^ No specific risk factor for these recurrent cases was identified.

Knotted stents have been removed using retrograde endoscopic, antegrade percutaneous and open approaches. The most common approach is retrieving the distal end of the stent and applying gentle continuous traction via a retrograde endoscopic approach.^3^ Ureteroscopy and laser to the knotted segment has also been described.^2^ Sighinofil et al. described using continuous catheter traction after failed ureteroscopic extraction due to a tight ureter. The stent was placed on traction using a catheter tape and removed after 3 continuous days of gentle traction. Three reports described untying the knot in situ by a retrograde approach - 2 using a guide wire through the stent and one using 5Fr alligator forceps to untie the knot.

Seven cases of antegrade removal of a knotted stent via percutaneous techniques have been described.^2^ In 5 of these, the stent was able to be relatively easily removed due to a dilated proximal ureteric segment. Kim et al. reported forming a loop using a 0.018 Terumo wire and catching the knotted stent with the folded wire to remove it.^4^ In our reported case, we removed the stent with an antegrade approach using snares and no nephroscopy. This is a useful approach to minimize intrarenal pressures in the setting of obstructive urosepsis.

## 4 Conclusion

Knotted stents are a rare and difficult complication to manage for urologists. A high index of suspicion is required if resistance is encountered on removal. We have described a percutaneous one stage approach for antegrade stent removal, in the context of obstruction and urinary sepsis, which is useful for urologists to be familiar with if faced with this clinical dilemma.

## Funding

This research did not receive any specific grant from funding agencies
